# Transcriptional responses in *Parascaris univalens* after *in vitro* exposure to ivermectin, pyrantel citrate and thiabendazole

**DOI:** 10.1186/s13071-020-04212-0

**Published:** 2020-07-09

**Authors:** Frida Martin, Faruk Dube, Oskar Karlsson Lindsjö, Matthías Eydal, Johan Höglund, Tomas F. Bergström, Eva Tydén

**Affiliations:** 1 Division of Parasitology, Department of Biomedical Sciences and Veterinary Public Health, Swedish University of Agricultural Sciences, Box 7036, 750 07 Uppsala, Sweden; 2 SLU-Global Bioinformatics Centre, Department of Animal Breeding and Genetics, Swedish University of Agricultural Sciences, Box 7023, 750 07 Uppsala, Sweden; 3 Institute for Experimental Pathology at Keldur, University of Iceland, Keldnavegur 3, 112 Reykjavik, Iceland; 4 Department of Animal Breeding and Genetics, Swedish University of Agricultural Sciences, Box 7023, 750 07 Uppsala, Sweden

**Keywords:** Transcriptome, Anthelmintic resistance, RNA sequencing, Differential expression, *lgc-37*

## Abstract

**Background:**

*Parascaris univalens* is a pathogenic parasite of foals and yearlings worldwide. In recent years, *Parascaris* spp. worms have developed resistance to several of the commonly used anthelmintics, though currently the mechanisms behind this development are unknown. The aim of this study was to investigate the transcriptional responses in adult *P. univalens* worms after *in vitro* exposure to different concentrations of three anthelmintic drugs, focusing on drug targets and drug metabolising pathways.

**Methods:**

Adult worms were collected from the intestines of two foals at slaughter. The foals were naturally infected and had never been treated with anthelmintics. Worms were incubated in cell culture media containing different concentrations of either ivermectin (10^−9^ M, 10^−11^ M, 10^−13^ M), pyrantel citrate (10^−6^ M, 10^−8^ M, 10^−10^ M), thiabendazole (10^−5^ M, 10^−7^ M, 10^−9^ M) or without anthelmintics (control) at 37 °C for 24 h. After incubation, the viability of the worms was assessed and RNA extracted from the anterior region of 36 worms and sequenced on an Illumina NovaSeq 6000 system.

**Results:**

All worms were alive at the end of the incubation but showed varying degrees of viability depending on the drug and concentration used. Differential expression (*P*adj < 0.05 and log2 fold change ≥ 1 or ≤ − 1) analysis showed similarities and differences in the transcriptional response after exposure to the different drug classes. Candidate genes upregulated or downregulated in drug exposed worms include members of the phase I metabolic pathway short-chain dehydrogenase/reductase superfamily (SDR), flavin containing monooxygenase superfamily (FMO) and cytochrome P450-family (CYP), as well as members of the membrane transporters major facilitator superfamily (MFS) and solute carrier superfamily (SLC). Generally, different targets of the anthelmintics used were found to be upregulated and downregulated in an unspecific pattern after drug exposure, apart from the GABA receptor subunit *lgc-37*, which was upregulated only in worms exposed to 10^−9^ M of ivermectin.

**Conclusions:**

To our knowledge, this is the first time the expression of lgc-37 and members of the FMO, SDR, MFS and SLC superfamilies have been described in *P. univalens* and future work should be focused on characterising these candidate genes to further explore their potential involvement in drug metabolism and anthelmintic resistance.
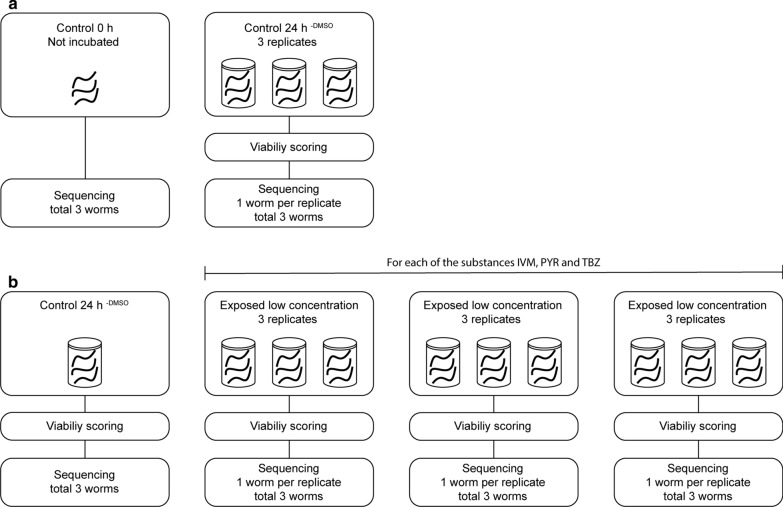

## Background

Nematodes within the genus *Parascaris* are pathogenic parasites of foals and yearlings worldwide. Traditionally the parasite has been referred to as *Parascaris equorum* but recent cytological studies have established that the major species currently infecting horses in the USA and Europe is *Parascaris univalens* [1–3]. *Parascaris* spp. infection causes nasal discharge, coughing and impaired growth, while large burdens can be lethal due to obstruction and perforation of the small intestine [4, 5]. To avoid parasite-related disease, most foals are usually treated with anthelmintics from the drug classes macrocyclic lactones, benzimidazoles or tetrahydropyrimidines several times during the first year [6]. Macrocyclic lactones act by binding to parasite-specific glutamate- and γ-aminobutyric acid (GABA) gated ion channels in nerve and muscle cells, increasing the cells permeability to Cl^-^ ions and leading to hyperpolarization which results in paralysis of the parasite [7]. Benzimidazoles bind parasite β-tubulin molecules, thereby disrupting the polymerisation of microtubules, causing starvation and death of the worm [8]. Tetrahydropyrimidines act as an agonist to the L-type nicotine acetylcholine gated ion channels, allowing Cl^−^ to flow through, leading to depolarisation of muscle cells and spastic paralysis of the parasite [9].

Overuse of anthelmintic drugs has contributed to the development of resistance in several parasites of veterinary importance [10]. The first reported case of anthelmintic resistance in *Parascaris* spp. was to the macrocyclic lactone ivermectin in 2002 [11] and since then ivermectin resistance has been reported from around the world and is now considered widespread [12]. Resistance to the tetrahydropyrimidine pyrantel was first discovered in the USA in 2008 [13] and has also been found in Australia [14] and, more recently, in Europe [3, 15]. The benzimidazole fenbendazole is generally effective against *Parascaris* spp. in Europe [3, 15], but sporadic cases of treatment failure have been reported from Australia [14] and Saudi Arabia [16]. Considering the risk of lethal complications in foals infected with *Parascaris* spp. and the lack of new anthelmintic drugs for the equine market, the development of resistance to all available drug classes in *Parascaris* spp. is a major threat to equine health and the equine industry.

Despite the increasing problem of anthelmintic resistance, the underlying causes of the development of resistance in parasitic nematodes are still poorly understood, particularly in ascarids. Advances in molecular techniques and genetics, such as the recent publication of a draft genome of *P. univalens* [17], have provided new possibilities to investigate responses in potential drug targets and pathways that may lead to resistance. There are several suggested mechanisms of anthelmintic resistance, including conformational changes or altered expression of the target molecule of the drug. Examples of conformational changes in the drug target are the three single nucleotide polymorphisms (SNPs) in the β-tubulin gene of strongyle nematodes. These mutations lead to amino acid substitutions and have been associated with the loss of action of benzimidazoles, particularly in strongyle nematodes of veterinary importance [18–20]. Decreased sensitivity to macrocyclic lactones in *Haemonchus contortus* was suggested to be connected to SNPs in the GABA receptor subunit gene *lgc-37* [21, 22], although this was challenged [23]. Additionally, reduced expression of the glutamate gated chloride channel *avr-14* was described in ivermectin resistant isolates of *Cooperia oncophora* and *Ostertagia ostertagi* [24].

Other proposed mechanisms of anthelmintic resistance include changes in drug metabolising enzymes and alterations in efflux pumps leading to inactivation or removal of the drugs [25]. Drug metabolism is usually divided into two phases; in phase I oxidation, reduction or hydrolysis convert the drug to a more reactive compound that can be conjugated with an endogenous molecule such as glutathione or glucose in phase II. This results in a soluble, inactive drug that can be removed from the cell [26]. The phase I enzymes of the cytochrome P450-family (CYP) have been shown to be involved in drug resistance in insects [27], but the involvement of phase I and II enzymes in anthelmintic resistance of parasitic nematodes has not been thoroughly investigated, particularly not in ascarids. However, constitutively higher expression of a CYP34/35 family member [28] as well as the phase II enzyme uridine 5′-diphospho-glucuronosyltransferase-glucosyltransferase (UGT) [29] have been found in BZ resistant strains of *H. contortus*. Several studies have also shown evidence for upregulation of genes encoding drug efflux pumps, such as ATP-binding cassette (ABC) transporters, in macrocyclic lactone resistant strongyle nematodes [30–32]. Taken together, these results indicate that changes in drug metabolism and efflux, play a role in anthelmintic resistance.

Despite anthelmintic resistance in *P. univalens* being a growing threat to equine health, little is known about the molecular mechanisms behind the development of resistance. To our knowledge, no *in vivo* treatment experiments have been performed in this parasite to date and only two studies have reported the expression of genes after *in vitro* drug exposure of adult *Parascaris* spp. [33, 34].

The aim of our study was to identify genes that respond to drug treatment, focusing on drug targets, metabolising enzymes and transporters in adult *P. univalens*. We have used a whole transcriptome approach to compare the gene expression after *in vitro* exposure to sub-lethal doses of ivermectin, pyrantel citrate and thiabendazole.

## Methods

### Parasite material and karyotype

Adult *P. univalens* were collected from the intestines of two Icelandic foals, approximately six months-old at an abattoir in Selfoss, Iceland. The horses originated from the same farm in southern Iceland and had never been treated with anthelmintic drugs. After removal from the intestine, worms were rinsed with 37 °C PBS (Life Technologies, Carlsbad, USA) and transported to the laboratory (Institute for Experimental Pathology at Keldur, University of Iceland, Reykjavík, Iceland) in an insulated box. A faecal sample was taken from one of the foals for species identification by karyotyping of *Parascaris* spp. eggs as described in Martin et al. [3].

### *In vitro* incubation experiment

**Fig. 1:**
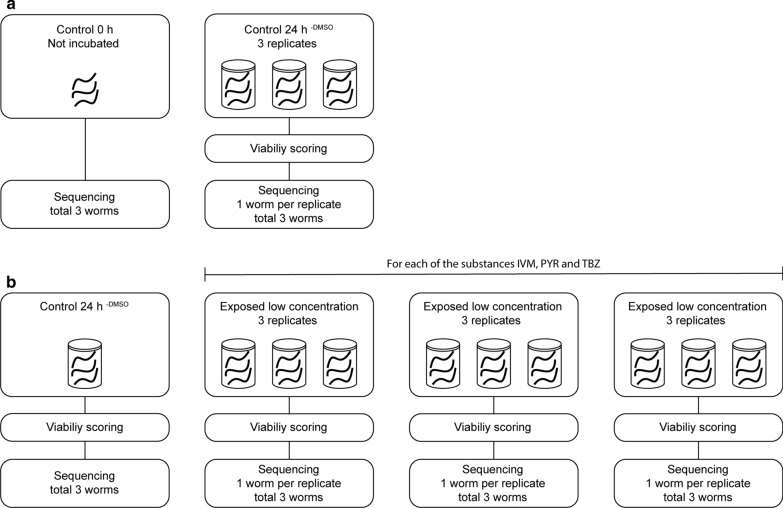
Overview of study design. In the *in vitro* incubation experiment (**a**) three individual *Parascaris univalens* worms were sequenced for the 0 h control. For the Control 24 h^−DMSO^, nine worms were incubated in cell culture media in three containers (three worms per container). One worm from each container was used for RNA sequencing, in total three worms. In the *in vitro* drug exposure experiment (**b**), three worms were incubated in cell culture media containing 0.1% DMSO (Control 24 h^+DMSO^) and used for RNA sequencing. For each of the three anthelmintic substances ivermectin (IVM), pyrantel citrate (PYR) and thiabendazole (TBZ), three different concentrations of the drugs were used (see Table 1). For each concentration, three worms were incubated in each of three different containers, in total nine worms for every concentration. From each container, one worm was used for RNA sequencing, in total three worms for every concentration. In total 36 worms were sequenced in this study

To investigate the effect of *in vitro* incubation on viability and gene expression, two groups of worms were used. One group of nine worms was divided into three containers containing cell culture media (RPMI-1640 with the addition of 10% foetal bovine serum, 1% penicillin/streptomycin and 1% L-glutamine (Life Technologies, Carlsbad, USA) and then incubated for 24 h at 37 °C (control 24 h^−DMSO^). The other group of worms (*n* = 3) was immediately killed by removal of the anterior region upon arrival to the laboratory to serve as controls for the *in vitro* incubation (control 0 h) (Fig. 1).

### *In vitro* drug exposure experiment

Worms were divided into three groups, one for each drug class. The worms were exposed to three different concentrations of ivermectin (IVM) (Sigma-Aldrich, Saint Louis, USA), pyrantel citrate (PYR) (Santa Cruz Biotechnology, Dallas, USA) or thiabendazole (TBZ) (Sigma-Aldrich), according to Table 1. The drug concentrations used were based on previous studies by Janssen et al. [34] for ivermectin exposure and Zhao et al. [35] for pyrantel citrate and thiabendazole exposure. All anthelmintic drugs were dissolved in DMSO (Swedish Veterinary Institute, Uppsala, Sweden) and serially diluted before addition to the media. For each concentration, three biological replicates, each consisting of three worms, were divided into different containers containing media and drug (final concentration of DMSO 0.1%) and incubated at 37 °C for 24 h. An additional group of three worms, serving as control, was incubated in media with the addition of 0.1% DMSO (control 24 h^+DMSO^) (Table 1, Fig. 1).

**Table 1:** Samples included in the *in vitro* exposure experiment

Drug and concentration	Viability	Differentially expressed genes	Unknown genes (%)
IVM 10^−9^ M	3.5	256	44
IVM 10^−11^ M	4.5	119	44
IVM 10^−13^ M	6.0	177	46
PYR 10^−6^ M	3.0	38	50
PYR 10^−8^ M	4.5	191	45
PYR 10^−10^ M	6.0	84	50
TBZ 10^−5^ M	4.0	154	46
TBZ 10^−7^ M	5.0	46	43
TBZ 10^−9^ M	5.0	161	51
Control 24 h^+ DMSO^	6.0	–	–

*Notes*: Mean viability score of adult *Parascaris univalens*, number of differentially expressed genes (adjusted *P* < 0.05 and log2 fold change of ≥ 1 or ≤ − 1) and proportion of unknown differentially expressed genes after exposure to the anthelmintic drugs ivermectin (IVM), pyrantel citrate (PYR) and thiabendazole TBZ), at different concentrations for 24 h. The gene expression of drug-exposed worms was normalized to control 24 h^+DMSO^

### Viability scoring and dissection

After 24 h incubation, worm viability was assessed according to the scoring system developed by Scare et al. [36] to evaluate the effects of the different drug concentrations on viability. The worms in each container were observed and viability scores between 2 (movement only when stimulated with forceps) and 6 (seven or more whole body movements without stimulation) were given for each container. One worm from each container (*n* = 3) was dissected and the anterior region, containing the pharynx and a small part of the anterior intestine, was placed in individual tubes containing RNAlater (Ambion). Three worms were dissected directly upon arrival at the laboratory, serving as controls for the *in vitro* incubation experiment (control 0 h). The samples were then transported to Sweden (Swedish University of Agricultural Sciences, Department of Biomedical Sciences and Veterinary Public Health, Division of Parasitology) for the molecular analysis.

### RNA extraction and sequencing

The samples were removed from RNAlater and cut into smaller pieces. One ml of Trizol (Invitrogen, Carlsbad, USA) was added to each sample and the tissue was homogenized in a glass tissue grinder. After addition of 0.2 ml of chloroform and centrifugation of the sample, 100 µl of the upper aqueous phase was mixed with 350 µl lysis buffer from the NucleoSpin® RNA Plus Kit (Macherey Nagel, Düren, Germany). RNA was then isolated according to the manufacturer’s instructions as described in the user manual of the kit. rDNase treatment and subsequent clean-up (NucleoSpin RNA Clean-up, Macherey Nagel) was performed according to the manufacturer’s protocol to ensure RNA purity.

Sample preparation and sequencing were performed at SciLifeLab Uppsala, SNP&SEQ Technology Platform. RNA concentration and integrity were checked by Fragment Analyzer (Agilent, Santa Clara, USA) before sequencing libraries were prepared from 500 ng total RNA using the TruSeq Stranded mRNA Library Preparation Kit including polyA selection (Illumina Inc., San Diego, USA) resulting in insert sizes of approximately 140 bp. Three biological replicates per condition were sequenced using Illumina NovaSeq S1 flow cells and 100 bp paired end v1 sequencing chemistry, resulting in 36 transcriptomes.

### RNA-seq analysis

Read quality assessment, adaptor removal, filtering and removal of duplicates was performed using fastp [37] (Pipeline with nextflow and docker support available at https://github.com/SLUBioinformaticsInfrastructure/RNAseq_nf.). The resulting reads were mapped against the predicted transcriptome of *P. univalens* (parascaris_univalens.PRJNA386823.WBPS11.mRNA_transcripts.fa) available in WormBase ParaSite (https://parasite.wormbase.org/Parascaris_univalens_prjna386823/Info/Index/) and transcripts were quantified using Salmon v.0.11.3 [38]. Transcript-level abundance, estimated counts and lengths from Salmon were summarised together with gene-ids from the *P. univalens* transcriptome into matrices by the R package *tximport* [39] for downstream differential expression analysis. Genes with five or more read counts were included in the differential expression analysis performed by the R package *DESeq2*, v.1.22.2 [40]. *P*-values resulting from the Wald test incorporated into *DESeq2* were adjusted for multiple testing using the Benjamini-Hochberg procedure [41] as applied by the R base p.adjust function. Genes with a log2 fold change of ≥ 1 or ≤ − 1 and an adjusted *P*-value < 0.05 were considered differentially expressed.

For principal components analysis (PCA), gene counts were transformed into log2 scale by applying regularized logarithm approach using the rlog function provided in the *DESeq2* package and plotted using the plotPCA function. For all the above analyses, R v.3.5.2 was used [42]. Functional annotations of differentially expressed genes were identified by searching the protein sequences against the Swiss-Prot database [43] using BlastP (e-value ≤ 10^−5^). Candidate genes for drug metabolism and drug targets significantly differentially expressed after exposure to one or more drugs were identified by their orthologue name. To identify the number of genes common among the concentrations of a particular drug, gene IDs were used to construct comparative Venn diagrams using the venn.diagram function of the *VennDiagram* package in R v.3.6.1 [44]. Gene IDs corresponding to individual Venn diagram partitions were retrieved using the get.venn.partition function. Superfamilies of genes shared between concentrations were identified by BlastP searches (e-value ≤ 10^−6^) in NCBI (https://www.ncbi.nlm.nih.gov/).

## Results

### Karyotype

*Parascaris univalens* was identified in the karyotype as DAPI stained eggs from the first mitotic division showed one pair of chromosomes.

### Viability after *in vitro* incubation

After 24 h incubation, all worms were alive irrespective of drug and concentration used for exposure. Control worms were highly viable (score 6), whereas worms incubated in the highest drug concentrations were visibly less viable (score 3–4) than those incubated in lower concentrations (score 5–6) (Table 1).

### Sequencing, quality control and mapping

RNA with RIN between 6.6–8.7 were sequenced on Illumina Nova Seq. The number of reads per sample varied between 63–132 million after quality assessment and filtering by fastp. Sequences have been deposited in the European Nucleotide Archive (ENA) under the accession number PRJEB37010 (https://www.ebi.ac.uk/ena). Mapping against the transcriptome resulted in an average mapping rate of 80–90% indicating high similarity between the transcriptomic data and the reference transcriptome.

### Analysis of differentially expressed genes

**Fig. 2:**
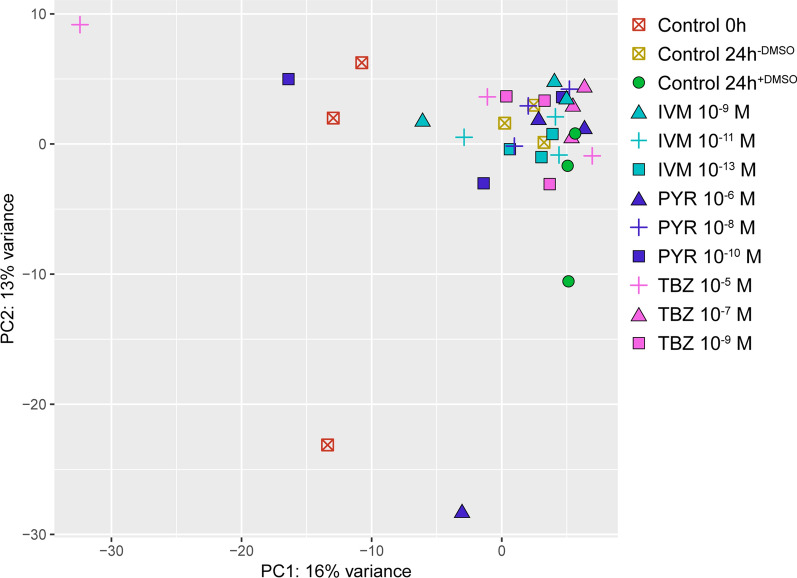
Principal components analysis plot showing the clustering of adult *Parascaris univalens* incubated in the presence of the anthelmintic drugs ivermectin (IVM), pyrantel citrate (PYR) and thiabendazole (TBZ) at different concentrations, as well as control worms. The largest variation in gene expression was observed between the non-incubated worms (control 0 h) and all worms incubated *in vitro*. Each symbol corresponds to an individual worm

The number of differentially expressed genes for each drug concentration and the proportion of uncharacterised genes are shown in Table 1. The PCA plot illustrates the differences in gene expression between individual worms (Fig. 2). It should be noted that the largest difference in gene expression was observed between the non-incubated worms (control 0 h) and both control and drug-exposed worms that were incubated *in vitro* for 24 h. Although care was taken to choose the largest worms (i.e. females) for the experiment, it was discovered during the dissection that two individuals (IVM 10^−11^ M individual 2 and TBZ 10^−5^ M individual 1) lacked egg-containing uterus. These two individuals did not show up as outliers in the PCA plot (Fig. 2) and were therefore included in the analysis. Log2 fold change and adjusted *P*-values for the differentially expressed genes discussed in this article are listed in Additional file 1: Table S1.

### Transcriptional response in control worms after *in vitro* incubation

The largest number of differentially expressed genes was seen in the *in vitro* incubation experiment, where unexposed worms incubated in media (control 24 h^−DMSO^) were compared to non-incubated worms (control 0 h). Between these two groups, 1061 genes were differentially expressed and interestingly, among these were a number of candidate genes putatively involved in drug metabolism, such as CYPs, flavin containing monooxygenase (FMO), short chain dehydrogenase/reductase (SDR), glutathione S-transferase (GST) and UGTs, as well as ABC-transporters (Additional file 2: Table S2). Forty percent of the differentially expressed genes were uncharacterised.

### Differential expression of putative drug targets

**Fig. 3:**
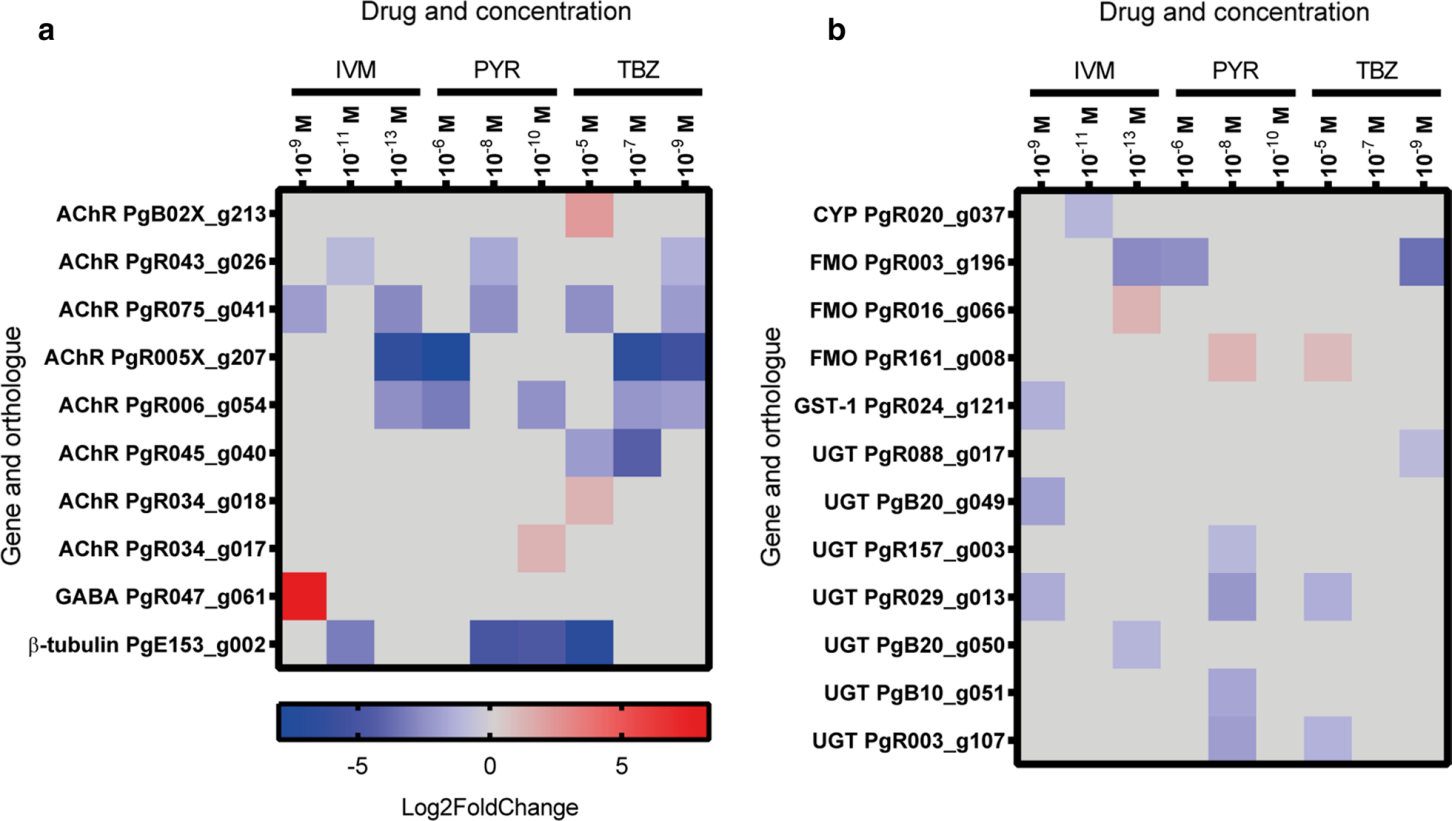
Heat map showing the differential expression of genes coding for putative drug targets (**a**) and genes coding for putative phase I and phase II metabolizing enzymes (**b**) after *in vitro* exposure of *Parascaris univalens* to the anthelmintic drugs ivermectin (IVM), pyrantel citrate (PYR) and thiabendazole (TBZ) at different concentrations. Genes with a log2 fold change of ≥ 1 or ≤ − 1 and an adjusted *P*-value (Walds test and Benjamini-Hochberg procedure) < 0.05 were considered differentially expressed

Putative drug targets were identified by their annotation and orthologue. Transcripts of ten drug targets were differentially expressed in worms exposed to IVM, PYR or TBZ compared to control 24 h^+DMSO^ (Fig. 3a). An orthologue to the GABA-receptor subunit *lgc-37* in *H. contortus* (GenBank: X73584), transcript PgR047_g061, was upregulated after exposure to the highest concentration of IVM (10^−9^ M). Three transcripts orthologous to acetylcholine receptor subunits (AChRs); PgB02X_g213, PgR034_g017 and PgR034_g018, were upregulated, whereas five AChRs; PgR043_g026, PgR075_g041, PgR005X_g207, PgR006_g054 and PgR045_g040, were downregulated according to Fig. 3a. The transcript PgE153_g002, identical to the previously described *P. equorum* β-tubulin isotype 2 (GenBank: KC713798), was downregulated after exposure to the highest dose of TBZ (10^−5^ M) but also after exposure to IVM (10^−11^ M) and PYR (10^−8^ M and 10^−10^ M) (Fig. 3a).

### Comparison of the transcriptional response after exposure to different concentrations of IVM, PYR or TBZ

The ten most upregulated and downregulated genes after exposure to each drug are shown in Table 2. Of the most upregulated genes across all drugs, 57% are uncharacterized and only one transcript, the GABA-receptor subunit PgR047_ g061 (*lgc-37*), has previously been described as a drug target to IVM and to be involved in anthelmintic resistance. Of the most downregulated genes after exposure to the three different drugs, 53% are uncharacterized and two transcripts, the acetylcholine receptor PgR005X_g207 and the B-tubulin isotype 2 gene PgE153_g002, are previously described as drug targets of PYR and TBZ. No genes previously described to be involved in drug metabolism were found in this list.

**Table 2:** The 10 most up- and downregulated genes (adjusted *P*-value < 0.05) in *Parascaris univalens* worms exposed to three different concentrations of the anthelmintic drugs ivermectin (IVM), pyrantel citrate (PYR) and thiabendazole (TBZ) for 24 h

Gene ID	Log2FC	Orthologue	Condition
PgR001X_g190	23.45	Uncharacterised	IVM 10^−9^ M
PgR013_g129	8.93	RPE Ribulose-phosphate 3-epimerase	IVM 10^−13^ M
PgR047_g061	8.23	GABRA6 Gamma-aminobutyric acid receptor subunit alpha-6	IVM 10^−9^ M
PgB04_g079	6.77	RPL3 60S ribosomal protein L3	IVM 10^−13^ M
PgR010_g071	5.77	Uncharacterised	IVM 10^−9^ M
PgR152_g010	5.36	COL9A1 Collagen alpha-1(IX) chain	IVM 10^−11^ M
PgR045_g001	5.32	Uncharacterised	IVM 10^−9^ M
PgR004_g064	4.85	Uncharacterised	IVM 10^−13^ M
PgR001X_g079	4.35	Uncharacterised	IVM 10^−9^ M
PgB05_g040	4.30	Uncharacterised	IVM 10^−13^ M
PgB04_g079	9.10/5.51	RPL3 60S ribosomal protein L3	PYR 10^−6^ M/PYR 10^−10^ M
PgE230_g001	8.25	Uncharacterised	PYR 10^−6^ M
PgR020_g019	6.13	Uncharacterised	PYR 10^−10^ M
PgR004_g204	5.88	Uncharacterised	PYR 10^−8^ M
PgR152_g010	5.67	COL9A1 Collagen alpha-1(IX) chain	PYR 10^−6^ M
PgR001X_g079	5.01	Uncharacterised	PYR 10^−8^ M
PgR099_g042	4.80	hpo-4 Putative GPI-anchor transamidase	PYR 10^−8^ M
PgE192_g001	3.71	Uncharacterised	PYR 10^−8^ M
PgR002_g035	3.63	ani-2 Anillin-like protein 2	PYR 10^−6^ M
PgR123_g005	3.48	Pym Partner of Y14 and mago	PYR 10^−8^ M
PgR307_g002	12.41/11.44	EDF1 Endothelial differentiation-related factor 1 homolog	TBZ 10^−9^ M/TBZ 10^−5^ M
PgR256X_g003	8.82	Uncharacterised	TBZ 10^−9^ M
PgR010_g071	8.22	Uncharacterised	TBZ 10^−9^ M
PgR190X_g006	8.03	Uncharacterised	TBZ 10^−9^ M
PgR001X_g019	7.85	Uncharacterised	TBZ 10^−7^ M
PgR152_g010	7.15	COL9A1 Collagen alpha-1(IX) chain	TBZ 10^−5^ M
PgB04_g079	6.49	RPL3 60S ribosomal protein L3	TBZ 10^−9^ M
PgR034_g082	6.33	Uncharacterised	TBZ 10^−9^ M
PgR099_g042	5.01	hpo-4 Putative GPI-anchor transamidase	TBZ 10^−7^ M
PgR001X_g079	4.76	Uncharacterised	TBZ 10^−9^ M
PgR166_g010	− 7.80/− 5.92	agmo-1 alkylglycerol monooxygenase	IVM 10^−13^ M/IVM 10^−9^ M
PgR049_g007	− 7.50	Uncharacterised	IVM 10^−13^ M
PgE193_g002	− 6.88	cyb-3 G2/mitotic-specific cyclin-B3	IVM 10^−9^ M
PgR260_g001	− 6.74	Eif4a3 Eukaryotic initiation factor 4A-III	IVM 10^−9^ M
PgR005X_g207	− 6.28	cup-4 Acetylcholine receptor-like protein cup-4	IVM 10^−13^ M
PgE182_g003	− 6.11	ZFP36 mRNA decay activator protein	IVM 10^−9^ M
PgR002_g265	− 6.02	Uncharacterised	IVM 10^−9^ M
PgR022X_g031	− 5.72	Uncharacterised	IVM 10^−13^ M
PgB32_g014	− 5.71	Uncharacterised	IVM 10^−9^ M
PgE068_g001	− 5.70	Uncharacterised	IVM 10^−9^ M
PgR022X_g031	− 8.43	Uncharacterised	PYR 10^−6^ M
PgB08_g035	− 8.13	ZC116.3 Probable cubilin	PYR 10^−8^ M
PgR389_g001	− 7.93	Uncharacterised	PYR 10^−8^ M
PgR005X_g207	− 7.90	cup-4 Acetylcholine receptor-like protein cup-4	PYR 10^−6^ M
PgR049_g007	− 7.84	Uncharacterised	PYR 10^−10^ M
PgB22_g038	− 6.34	F01G4.6 Phosphate carrier protein, mitochondrial	PYR 10^−10^ M
PgR022X_g030	− 5.72	Uncharacterised	PYR 10^−6^ M
PgB08_g034	− 5.65	ZC116.3 Probable cubilin	PYR 10^−6^ M
PgR166_g010	− 5.57	agmo-1 Alkylglycerol monooxygenase	PYR 10^−8^ M
PgR057_g068	− 5.51	Uncharacterised	PYR 10^−10^ M
PgR012_g016	− 13.72	Uncharacterised	TBZ 10^−9^ M
PgR049_g007	− 9.59/− 8.19	Uncharacterised	TBZ 10^−7^ M/TBZ 10^−9^ M
PgR004_g249	− 8.12	rps28 40S ribosomal protein S28	TBZ 10^−7^ M
PgB15_g052	− 6.84	Uncharacterised	TBZ 10^−9^ M
PgE153_g002	− 6.84	Tubulin beta-1 chain	TBZ 10^−5^ M
PgR022X_g030	− 6.83	Uncharacterised	TBZ 10^−9^ M
PgR011_g087	− 6.76	Uncharacterised	TBZ 10^−9^ M
PgE193_g002	− 6.70	cyb-3 G2/mitotic-specific cyclin-B3	TBZ 10^−5^ M
PgR022X_g031	− 6.46	Uncharacterised	TBZ 10^−9^ M
PgR005X_g207	− 6.32	cup-4 Acetylcholine receptor-like protein cup-4	TBZ 10^−7^ M

**Fig. 4:**
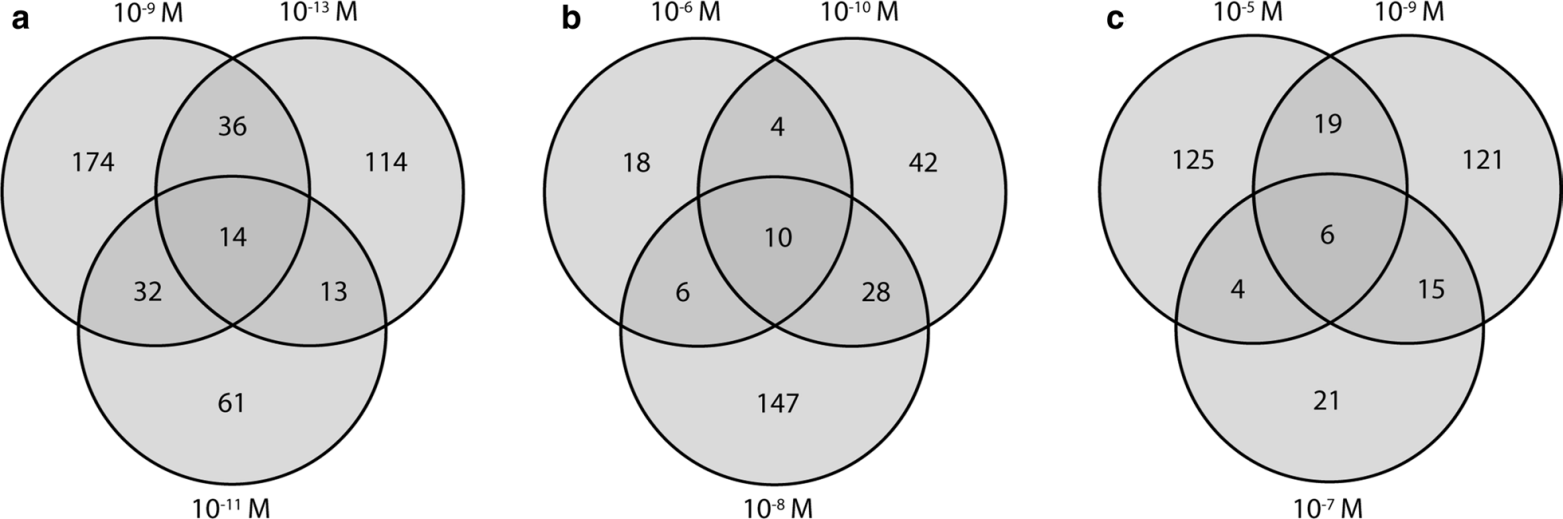
Venn diagrams showing the number of differentially expressed genes unique to and shared among *Parascaris univalens* worms exposed to three different concentrations of the anthelmintic drugs ivermectin (**a**), pyrantel citrate (**b**) and thiabendazole (**c**). Genes with a log2 fold change of ≥ 1 or ≤ − 1 and an adjusted *P*-value (Walds test and Benjamini-Hochberg procedure) < 0.05 were considered differentially expressed

Differentially expressed genes shared among the three concentrations of each drug are visualized in Fig. 4 and Table 3. After IVM exposure, 14 transcripts showed overlapping differential expression for all three concentrations (Fig. 4a). Of these, nine transcripts were upregulated whereas five were downregulated. The differentially expressed genes included four transcripts belonging to the SDR superfamily, putatively involved in phase I metabolism, two transcripts of the major facilitator superfamily (MFS), known to be involved in drug efflux and resistance in bacteria and yeast, and one transcript of the solute carrier superfamily (SCS), involved in transportation. Of the remaining seven differentially expressed transcripts, three do not have any known connection to drug metabolism or transport, while four transcripts are uncharacterised (Table 3).

**Table 3:** Differentially expressed genes (log2 fold change ≥ 1 or ≤ − 1 and adjusted *P*-value < 0.05) and their corresponding superfamilies shared among *Parascaris univalens* worms exposed to three different concentrations of the anthelmintic drugs ivermectin (IVM), pyrantel citrate (PYR) and thiabendazole (TBZ) for 24 h

Gene ID	Superfamily		Log2FC	
Ivermectin	Concentration	10^−9^ M	10^−11^ M	10^−13^ M
PgB01_g106	Short-chain dehydrogenases/reductases (SDR) cl25409	− 1.05	− 1.57	− 1.50
PgR004_g112	Short-chain dehydrogenases/reductases (SDR) cl25409	1.94	1.94	1.27
PgR007_g080	Short-chain dehydrogenases/reductases (SDR) cl25409	1.92	1.75	1.80
PgR127_g021	Short-chain dehydrogenases/reductases (SDR) cl25409	1.54	1.50	1.36
PgR006_g137	The major facilitator superfamily (MFS) cl28910	− 1.12	− 1.39	− 1.92
PgR015_g078	The major facilitator superfamily (MFS) cl28910	2.32	2.11	2.44
PgR011_g039	Solute carrier families 5 and 6-like superfamily (SLC) cl00456	1.15	1.29	1.47
PgR135_g007	C-type lectin (CTL) cl02432	1.06	1.29	1.28
PgR422_g001	RNA recognition motif (RRM) superfamily cl17169	1.62	1.63	1.99
PgR037_g063	Clc-like superfamily cl06205	1.72	1.84	1.39
PgB06_g058	No conserved domains detected	1.69	1.77	2.51
PgR061_g018	No conserved domains detected	− 1.55	− 1.84	− 2.14
PgR142_g012	No conserved domains detected	− 2.15	− 1.26	− 1.53
PgR011_g030	No conserved domains detected	− 1.04	− 1.40	− 1.79

Pyrantel	Concentration	10^−6^ M	10^−8^ M	10^−10^ M
PgR006_g137	Major facilitator superfamily cl28910	− 1.97	− 1.88	− 1.70
PgB08_g086	Alpha/beta hydrolases cl21494	− 3.89	− 4.04	− 2.64
PgB27X_g004	C-type lectin (CTL) cl02432	− 3.11	− 4.46	− 2.05
PgR050X_g009	Olfactomedin-like domain cl02549	− 3.20	− 3.62	− 1.62
PgB08_g034	CUB domain cl00049	− 5.65	− 4.90	− 1.13
PgE206_g001	No conserved domains detected	− 2.88	− 3.92	− 3.08
PgR022X_g030	No conserved domains detected	− 5.72	− 5.15	− 2.92
PgR022X_g031	No conserved domains detected	− 8.43	− 4.43	− 2.23
PgR093_g009	No conserved domains detected	− 4.87	− 4.88	− 2.64
PgR186_g008	No conserved domains detected	1.05	1.82	1.51

Thiabendazole	Concentration	10^−5^ M	10^−7^ M	10^−9^ M
PgB08_g034	CUB domain cl00049	− 2.82	− 3.52	− 5.09
PgR004_g040	Carbonic anhydrase alpha (vertebrate-like) group cl00012	− 1.70	− 3.65	− 1.91
PgE192_g001	No conserved domains detected	1.30	3.48	2.17
PgR004_g250	No conserved domains detected	− 1.50	− 1.73	− 1.81
PgR022X_g030	No conserved domains detected	− 4.64	− 5.65	− 6.83
PgR049_g007	No conserved domains detected	− 6.11	− 9.59	− 8.19

*Note*: The gene expression of drug-exposed worms was normalized to control 24 h^+ DMSO^

After exposure to PYR, ten transcripts were commonly differentially expressed (Fig. 4b) and all but one were downregulated. As in the IVM exposed worms, the transcript PgR006_g137, a member of MFS, was downregulated (Table 3). Of the remaining differentially expressed transcripts, four belong to superfamilies not connected to drug metabolism or transportation, whereas five transcripts are uncharacterised (Table 3).

Six transcripts were commonly differentially expressed after exposure to all three concentrations of TBZ (Fig. 4c), five downregulated and one upregulated. Of these, two transcripts are involved in processes not connected to drug metabolism or transport, while four are uncharacterised or not similar to any known superfamily (Table 3).

### Differential expression of other putative candidate genes

Putative candidate genes involved in drug metabolism were identified by their annotation and orthologue. Transcripts of 12 metabolising genes involved in phase I and phase II drug metabolism were identified in worms exposed to all drugs (Fig. 3b).

Four phase I transcripts were differentially expressed after drug exposure. One transcript belonging to the CYP family (PgR020_g037) was downregulated after exposure to the medium concentration of IVM (10^−11^ M). Three transcripts belonging to the flavin containing monooxygenase (FMO) superfamily were differentially expressed in an inconsistent pattern after drug exposure. The transcript PgR003_g196 was downregulated after exposure to IVM (10^−9^ M), TBZ (10^−5^ M) and PYR (10^−10^ M). The transcript PgR016_g066 was upregulated after exposure to IVM (10^−9^ M) only, whereas the transcript PgR161_g008 was upregulated after exposure to PYR (10^−8^ M) and TBZ (10^−9^ M) (Fig. 3b).

Eight genes belonging to two superfamilies of the phase II metabolizing pathway were also differentially expressed in an inconsistent pattern irrespective of drug and concentration used (Fig. 3b). One transcript, PgR024_g121, of the glutatione S-transferase superfamily (GST), was down regulated, but only after exposure to the lowest concentration of IVM (10^−13^ M). Seven transcripts of the uridine 5′-diphospho-glucuronosyltransferase superfamily (UGT) were downregulated in an unspecific pattern after exposure to all drugs (Fig. 3b).

## Discussion

The equine roundworm *Parascaris* spp. is resistant to several classes of anthelmintic drugs, but regardless of this, few studies focusing on the mechanisms involved have been published [33, 34, 45]. In this study, the transcriptomes of 36 individual *P. univalens* worms, confirmed by karyotyping, were analysed after *in vitro* exposure to different concentrations of IVM, PYR and TBZ. We found, to our knowledge for the first time in ascarid worms, that the expression of phase I gene families SDR and FMO were affected by exposure to anthelmintic drugs. We also found a 250-fold upregulation of a GABA receptor subunit.

The GABA receptor subunit PgR047_g061, orthologous to the *H. contortus* gene *lgc-37*, was upregulated after exposure to the highest IVM concentration (10^−9^ M) (Fig. 3). GABA receptors are targets for IVM in nematodes, but generally show lower affinity to the drug than the glutamate receptors [22]. Even so, mutations in *lgc-37* have been found to decrease the sensitivity of macrocyclic lactone drugs in *H. contortus* [21] suggesting a role in resistance. Transcriptional regulation of *lgc-37* (PgR047_g061) in association with IVM response has not been reported previously, but taken together these results indicate that this gene could be an interesting candidate for further research in *P. univalens*.

Eight transcripts orthologous to AChR subunits, the putative drug target for PYR, were differentially expressed, but with no clear pattern after exposure to IVM, PYR and TBZ. Since the L-subtypes of AChRs have not yet been characterized in *Parascaris* spp., we cannot conclude whether these transcripts are the drug targets for pyrantel. Previous studies have shown that truncated forms of AChRs, as well as reduced expression of AChR subunits, have been associated with decreased sensitivity to PYR in strongyle nematodes [46, 47].

Furthermore, β-tubulin isotype 2 was downregulated after exposure to all three drug classes, however to a higher degree after TBZ exposure than after exposure to IVM or PYR. So far only two β-tubulin isotypes (isotype 1 and 2) have been described in *Parascaris* spp. [48], though more might be present in the genome since six putative β-tubulin genes were found in the closely related parasite *Ascaridia galli* [49]. It has also been mentioned that the *Ascaris suum* genome contains at least nine β-tubulin genes [50]. It should be noted that the isoforms of ascarid β-tubulins should not be directly compared to the strongyle nematodes as phylogenetic studies have shown that they are evolutionary separated [48, 49]. In a study by Martis et al. [49], where the transcriptomes of *A. galli* were compared before and after *in vivo* exposure to flubendazole, no differential expression of β-tubulins was found, whereas a study by Tyden et al. [51] showed an upregulation of β-tubulin isotype 1 in *Parascaris* spp. eggs exposed to thiabendazole during embryogenesis. However, downregulation of specific isotypes of β-tubulins has been observed in human cancer cells resistant to microtubule destabilising drugs [52, 53], indicating that expressional regulation of β-tubulin could be a cellular response to certain drugs.

Comparing the differential expression after exposure to three different drugs provided valuable insights on how expression of putative drug targets are similarly affected regardless of the drug used. Another point to consider is the inconsistent expression of putative drug targets and metabolising enzymes across the different concentrations of a drug. This could be explained by individual variation in gene expression between the biological replicates or can be an effect of changes in gene expression from drug metabolism to severe stress or apoptosis in response to high doses of anthelmintics. These are important findings to consider in the analysis of expression data and, to our knowledge, this is the first time this has been shown in *P. univalens*.

Several members of the phase I superfamily SDR were found to be differentially expressed after drug exposure in our study. The SDR family is a large superfamily present in all life forms [54], containing enzymes with conserved structure that metabolise endogenous and xenobiotic compounds by phase I reduction [55, 56]. Upregulation of SDR enzymes is believed to contribute to resistance towards chemotherapeutic agents in cancer treatment in human medicine [57] and has also been seen in *Caenorhabditis elegans* after *in vitro* exposure to benzimidazole drugs [58]. The involvement of SDR in drug metabolism in parasitic nematodes is largely unknown. It has been shown that reduction is the main metabolic pathway for flubendazole metabolism in *H. contortus*, though it has not been established if the enzymes involved belonged to the SDR family or other related superfamilies [59]. In summary, members of the SDR family may participate in the xenobiotic metabolism in helminths, but their possible involvement in anthelmintic resistance has not been studied in parasitic nematodes.

Members of the FMO family are phase I enzymes known to be involved in the biotransformation of xenobiotic compounds in many phyla [60]. FMO enzymes have been shown to play a role in albendazole metabolism in the common liver fluke *Fasciola hepatica* and to be involved in the resistance to this drug [61, 62]. In contrast, Vokřál et al. [63] did not observe any activity of FMO enzymes in *H. contortus* in response to albendazole. In our study, two transcripts of the FMO family were upregulated after exposure to all drugs, indicating that they may play a universal role in the drug metabolism in *P. univalens*.

Only one member of the CYP family (PgR020_g037) was differentially expressed in this study, with a downregulation after exposure to IVM (10^−11^ M). This finding is in contrast to the upregulation of the CYP family member PgR071_g005 in *P. univalens in vitro* exposed to IVM and oxibendazole [33]. Upregulation of CYP genes has also been observed in susceptible strains of *H. contortus* after *in vitro* exposure to IVM [26].

In summary, we found that several phase I enzymes were differentially expressed in *P. univalens* after drug exposure. To our knowledge, SDR and FMO enzymes have not yet been characterized in parasitic helminths and therefore their roles in drug metabolism or anthelmintic resistance have not been investigated. Although no consistent pattern was observed regardless of drug and concentration used, we found downregulation of several members of the phase II enzymes of the UGT family. In contrast, several studies have shown an increased expression and activity of UGTs in benzimidazole-resistant strains of *H. contortus* [29, 63]. Although the expression and function of phase I and phase II enzymes have been investigated in parasitic helminths, few studies have focused on ascarid worms and thus further investigation is required.

The upregulation of genes encoding transporters and drug efflux pumps have been suggested as a mechanism behind multidrug resistance in parasitic worms [30–32]. Focus has so far been mainly on certain ABC-transporters genes, but in agreement with Janssen et al. [34], we did not observe any changes in gene expression of *pgp-11* and *pgp-16* after drug exposure. On the other hand, we found that transcripts of the transportation superfamilies MFS and SCS were differentially expressed after exposure to anthelmintic drugs. These are large superfamilies of membrane proteins present in both prokaryotes and eukaryotes, transporting endogenous substances and drugs across cell membranes [64, 65]. The role of MFS proteins in drug resistance in parasitic helminths has so far not been investigated. However, their involvement in resistance to specific drugs, as well as multidrug resistance in bacteria and yeast, are well studied [64], indicating that these could be interesting candidates to investigate further in regards to anthelmintic resistance.

Among the ten most upregulated and downregulated genes after exposure to each of the three drugs, there were no previously described genes encoding xenobiotic enzymes or transporters. Even though the function of more than 50% of these genes is unknown, this list might contain novel candidates for drug metabolism and needs to be further investigated.

In a recently published study, Scare et al. [33] studied the transcriptomes of adult *P. univalens* after *in vitro* exposure to ivermectin and oxibendazole. Genes identified as potentially involved in drug detoxification and regulatory mechanisms differed from our results with only one similarity between the two studies, a member of the CYP family. These varying results are most likely due to the differences in study design between the two experiments, such as different drug concentrations and drug exposure times and the use of different tissues for RNA extraction. Scare et al. [33] also allowed a 24 h acclimatization period for the worms in tissue culture media before drug exposure, whereas we started the drug exposure at 0 h. These two studies show that despite similar aims, experimental design can affect differential expression considerably and thus also highlight the potential for future studies to identify important genes involved in drug interactions not found in either study.

In our experiment, unexposed control worms (control 24 h^−DMSO^) were not visibly affected by incubation at 37 °C for 24  h. Even so, we observed a large number (*n* = 1061) of differentially expressed genes in these worms when compared to non-incubated worms (control 0 h). Among these genes were several candidates putatively involved in drug metabolism and efflux. This implies that although not visibly affected by the *in vitro* incubation, the worms become stressed from the change in environment and nutritional supply when removed from the host intestine to incubation in media. This is an important point to consider when performing *in vitro* experiments. Although we cannot be sure that the *in vitro* response to drug treatment reflects what would happen in a similar *in vivo* situation, it is a way to obtain more knowledge about the parasite response to drug exposure. Since whole live animal experiments in foals are both expensive and pose ethical dilemmas, further development of *in vitro* models is crucial for research in parasitic nematodes.

## Conclusions

We have explored the changes in gene expression across the complete transcriptome of adult *P. univalens* in response to *in vitro* exposure of the anthelmintic drugs ivermectin, pyrantel citrate and thiabendazole. We found a 250-fold upregulation of a possible target of ivermectin, an orthologue to the GABA receptor subunit *lgc-37*, which is an interesting candidate gene to investigate further. Surprisingly, few other candidate genes found in other parasitic worms, such as *P-gps*, were differentially expressed in our experiment. However, our data revealed differential expression of several novel gene candidates belonging to the phase I superfamilies SDR and FMO, as well as transporters belonging to the MFS and SLC families. To our knowledge, this is the first time the expression of these genes has been described in *P. univalens* and future work should be focused on characterising these candidate genes further to explore their potential involvement in drug metabolism and anthelmintic resistance.

## Supplementary information

**Additional file 1: Table S1.** Log2 fold change and adjusted *P*-values for the differentially expressed genes discussed in this article.

**Additional file 2: Table S2.***Parascaris univalens in vitro* incubation experiment. Differentially expressed genes (fold change ≥ 2 or and adjusted *P*-value (Walds test and Benjamini-Hochberg procedure) < 0.05) and their corresponding orthologues according to Swiss-Prot database after 24 h *in vitro* incubation in media (control 24 h^-DMSO^). Gene expression was normalized to non-incubated worms (control 0 h).

## Data Availability

Nucleotide sequence data reported in this paper are available in the European Nucleotide Archive (ENA) under the accession number PRJEB37010 (https://www.ebi.ac.uk/ena).

## Abbreviations

SDR: short-chain dehydrogenase/reductase superfamily
FMO: monooxygenase superfamily
CYP: cytochrome P450-family
MFS: major facilitator superfamily
SLC: solute carrier superfamily
GABA: γ-aminobutyric acid
SNP: single nucleotide polymorphisms
PBS: phosphate-buffered saline
UGT: uridine 5′-diphospho-glucuronosyltransferase-glucosyltransferase
ABC: ATP-binding cassette
IVM: ivermectin
PYR: pyrantel
TBZ: thiabendazole
DMSO: dimethyl sulfoxide
PCA: principal components analysis
DAPI: 4’,6-diamidino-2-phenylindole
RIN: RNA integrity number
GST: glutathione S-transferase
AChRs: acetylcholine receptor subunits
